# *Burkholderia pseudomallei* distribution in Australasia is linked to paleogeographic and anthropogenic history

**DOI:** 10.1371/journal.pone.0206845

**Published:** 2018-11-05

**Authors:** Anthony L. Baker, Talima Pearson, Jason W. Sahl, Crystal Hepp, Erin P. Price, Derek S. Sarovich, Mark Mayo, Apichai Tuanyok, Bart J. Currie, Paul Keim, Jeffrey Warner

**Affiliations:** 1 Tasmanian Institute of Agriculture (TIA), University of Tasmania, Sandy Bay, Tasmania, Australia; 2 College of Public Health, Medical and Veterinary Sciences, James Cook University, Townsville, Queensland, Australia; 3 Pathogen and Microbiome Institute, Northern Arizona University, Flagstaff, Arizona, United States of America; 4 Informatics and Computing, Northern Arizona University, Flagstaff, Arizona, United States of America; 5 Global and Tropical Health Division, Menzies School of Health Research, Charles Darwin University, Darwin, Northern Territory, Australia; 6 Faculty of Science, Health, Education and Engineering, University of the Sunshine Coast, Sippy Downs, Queensland, Australia; 7 College of Veterinary Medicine, University of Florida, Gainesville, Florida, United States of America; Tulane University School of Medicine, UNITED STATES

## Abstract

*Burkholderia pseudomallei* is the environmental bacillus that causes melioidosis; a disease clinically significant in Australia and Southeast Asia but emerging in tropical and sub-tropical regions around the globe. Previous studies have placed the ancestral population of the organism in Australia with a single lineage disseminated to Southeast Asia. We have previously characterized *B*. *pseudomallei* isolates from New Guinea and the Torres Strait archipelago; remote regions that share paleogeographic ties with Australia. These studies identified regional biogeographical boundaries. In this study, we utilize whole-genome sequencing to reconstruct ancient evolutionary relationships and ascertain correlations between paleogeography and present-day distribution of this bacterium in Australasia. Our results indicate that *B*. *pseudomallei* from New Guinea fall into a single clade within the Australian population. Furthermore, clades from New Guinea are region-specific; an observation possibly linked to limited recent anthropogenic influence in comparison to mainland Australia and Southeast Asia. Isolates from the Torres Strait archipelago were distinct yet scattered among those from mainland Australia. These results provide evidence that the New Guinean and Torres Strait lineages may be remnants of an ancient portion of the Australian population. Rising sea levels isolated New Guinea and the Torres Strait Islands from each other and the Australian mainland, and may have allowed long-term isolated evolution of these lineages, providing support for a theory of microbial biogeography congruent with that of macro flora and fauna. Moreover, these findings indicate that contemporary microbial biogeography theories should consider recent and ongoing impacts of globalisation and human activity.

## Introduction

Melioidosis is a severe infection caused by the environmental saprophyte *Burkholderia pseudomallei* and is considered an emerging disease threat throughout tropical and sub-tropical regions globally. In endemic regions the bacterium can be isolated from soil and various freshwater sources [1–4]. Isolates of *B*. *pseudomallei* from these regions have been extensively characterized using multi-locus sequence typing (MLST), which has demonstrated that mainland Australian isolates are distinct to those from Asia [5–8]. Furthermore, whole genome based phylogenies of Australian isolates in comparison to those from Southeast Asia indicate that the organism most likely originated in Australia before being introduced into Southeast Asia in a single or limited introductory event [7, 9–11]. High genetic diversity in both Australian and Southeast Asian isolates indicate that this dispersion event took place long before present, yet there is very little evidence of admixture between populations. These observations indicate that biogeographical boundaries inhibiting both regional and long-range dispersal of the organism are strong.

**Fig 1:**
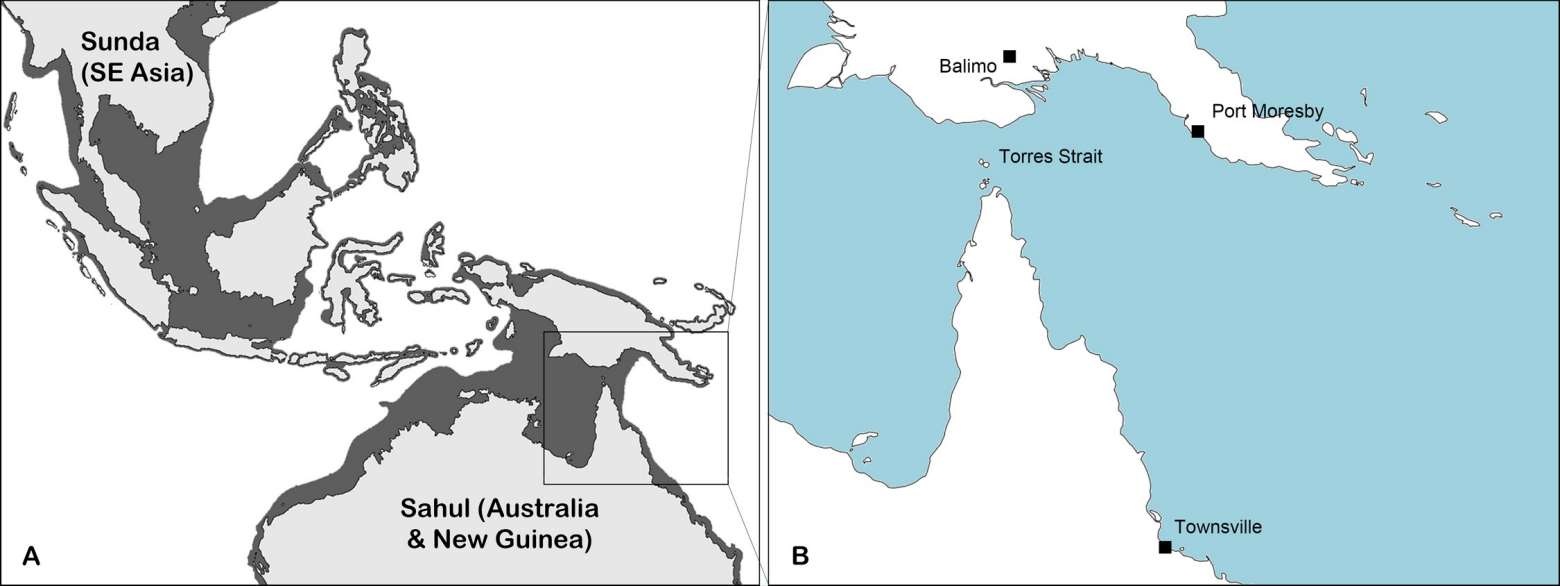
Map of Australasia and Southeast Asia. a) 21,500 years ago during the last glacial maximum. The shaded regions represent what was dry land during the period. Note that Australia and PNG comprised a single continent (Sahul) and that most of Southeast Asia (Sunda) was linked by land bridges. b) Present day locations of significance for this study. The Torres Strait archipelago is comprised of some 274 individual islands scattered between New Guinea and mainland Australia.

The Wallace Line [12] is a transitional area between Asian and Australian ecozones and has been hypothesized to represent the boundary between Asian and Australian *B*. *pseudomallei* populations [7, 13]. The boundary is based on a deep-water trench that persisted even during the lower sea levels of past glacial periods when much of mainland Asia was united with Indonesia and the Philippines to form the Sunda continent. Similarly, mainland Australia, the Torres Strait and New Guinea united to form the continent of Sahul which remained separated from Sunda by the deep Java and Philippine oceanic trenches [14, 15] (Fig 1). The degree of population isolation and the extent to which the Wallace Line defines the boundary between Australian and Asian *B*. *pseudomallei* populations is not well defined as few isolates from Indonesia, the Torres Straits, and New Guinea have been characterized. Addressing this question will improve our understanding of *B*. *pseudomallei* dispersal mechanisms, the potential role of humans and/or animals in its dissemination, and of geographical boundaries that have prevented gene flow between these populations.

Melioidosis has never been reported from the western half of New Guinea (Papua, Indonesia) and is only seldom reported from the east (Papua New Guinea), most likely as a result of poor primary healthcare in regional areas [16]. Although it is likely that the organism is widespread throughout lowland regions of the island, only two regions of melioidosis endemicity have been documented: Balimo in the Western Province and Port Moresby in the Central Province. As such, *B*. *pseudomallei* isolates from the island have only rarely been characterized [17]. We have previously analysed *B*. *pseudomallei* isolates from the Balimo region of New Guinea, where we identified a low level of genetic diversity [17]. Multi-locus variable-number tandem repeat analysis (MLVA) was used to hypothesize a long-term isolated environmental persistence of *B*. *pseudomallei* in the region that is subjected to limited anthropological influences [13]. Broader questions regarding the establishment of an isolated *B*. *pseudomallei* population in the region and their deeper evolutionary origins in a global context remain to be answered. Given the non-random distribution of *B*. *pseudomallei* genotypes throughout the adjacent Torres Strait archipelago that is indicative of island biogeographical influences and limited dispersal across a marine environment [18], it is likely that the paleogeography of the region is reflected in the present day *B*. *pseudomallei* population distribution.

Therefore, the aim of this study was to determine the evolutionary relationships among *B*. *pseudomallei* isolates from New Guinea, the Torres Strait region of northern Queensland and mainland Australia. Moreover, by comparing these isolates to those previously characterised from around the globe, we aim to progress our understanding of the dispersal and biogeography of melioidosis on a global scale.

## Materials and methods

### Ethics statement

Approval and ethical clearance for this study was granted by the Medical Research Advisory Committee (MRAC) of Papua New Guinea under MRAC No 10.03. All clinical isolates collected originate from diagnostic specimens, and as such patients did not provide written informed consent. MRAC is the appropriate body in Papua New Guinea to grant approval for the later use of clinical samples in research.

### Bacterial isolates

This study analysed 12 clinical (from individual patients) and two environmental isolates of *B*. *pseudomallei* (Table 1), four of which were retrieved from the Balimo region of Papua New Guinea as previously described [17]; five from patients at the Port Moresby Hospital [16, 19], whilst five clinical isolates were from the Torres Strait and the neighbouring region of far northern Queensland [18] (Fig 1). An additional seven isolates from the Townsville region of northern Queensland (TSV17, TSV24, TSV28, TSV30, TSV36, TSV44, TSV51) were sequenced for this study. Whole genome sequences for comparison were sourced online including an additional 19 isolates from mainland Australia, 31 isolates from Southeast Asia and ten isolates from broader regions of the globe. Isolates subject to whole genome sequencing (WGS) from Balimo were chosen based on MLST and MLVA data to include representatives providing the broadest diversity [13]. Remaining isolates were analysed with MLST to ensure that sequenced isolates were selected to best capture existing genetic diversity. Bacteria were stored in the James Cook University and Menzies School of Health Research culture collections at -80°C. Isolates were plated onto Ashdown’s agar [20] formulated in house and cultivated at 37°C for 48 hours prior to DNA extraction.

**Table 1:** *Burkholderia pseudomallei* isolates from Papua New Guinea and the Torres Strait sequenced for this study.

ID	Location	Year	Source	MLST	SRA Number
C12	Balimo, Western Province, PNG	2005	Clinical	668	SRR2896252
AG57	Adiba, Western Province, PNG	2001	Environmental	667	SRR2896253
C2	Kimama, Western Province, PNG	1995	Clinical	267	SRR2921951
K41	Kimama, Western Province, PNG	1998	Environmental	267	SRR2896254
MSHR0139	Port Moresby, Central Province, PNG	1987	Clinical	246	SRR2896255
MSHR0141	Port Moresby, Central Province, PNG	1992	Clinical	274	SRR2896256
MSHR1950	Port Moresby, Central Province, PNG	2005	Clinical	340	SRR2896271
POM1	Port Moresby, Central Province, PNG	2002	Clinical	248	SRR2896258
POM2	Port Moresby, Central Province, PNG	2001	Clinical	611	SRR2896259
TSI15	Boigu Island, Australia	2006	Clinical	598	SRR2896260
TSI19	Mabuiag Island, Australia	2002	Clinical	237	SRR2896261
TSI29	Laura, Australia	2004	Clinical	604	SRR2896262
TSI31	Thursday Island, Australia	2005	Clinical	606	SRR2896263
TSI32	Yam Island, Australia	2000	Clinical	610	SRR2896264

### DNA extraction and qualification

A single, large colony from each plate was removed to 1.5 ml O-ring screw-top microcentrifuge tubes (Sarstedt, Germany) containing 500 μl of Lysis buffer (Corbett Robotics, Australia) and 50 μg of proteinase K (Sigma, Australia). Tubes were incubated at 55°C for two hours in a Hybaid Shake 'n' Stack hybridization oven (Thermo Electron Corporation, MA, USA) with the rotisserie set to the lowest setting. Following lysis, tubes were supplemented with 250 μl of 10M guanidine hydrochloride then passed through Promega Wizard SV Genomic DNA Purification System (Promega, Australia) spin columns with subsequent purification as per the manufacturer’s directions. Quality and quantity of DNA was determined by NanoPhotometer (Implen, Germany).

### Multi-locus sequence typing

Isolates were screened for diversity using MLST. Pre-sequencing PCRs were performed in 200 μl thin walled PCR tubes (Sarstedt, Germany) using standard reagents from Promega (Australia) and contained: 1 × GoTaq colourless master mix (Promega), 0.8 μM mixed primers and molecular biology grade H_2_O (Sigma, Australia) to 30 μl. Primers for MLST were as described [21] with the recommended amendments listed on the *B*. *pseudomallei* MLST website (pubmlst.net/bpseudomallei). Cycling conditions consisted of an initial denaturation period of 3 min at 95°C, followed by 40 cycles of 95°C for 30 sec, 62°C for 30 sec and 72°C for 30 sec and a final elongation of 72°C for 10 min. Sequencing products were analysed by electrophoresis using a 1.5% agarose gel to ascertain correct fragment size, concentration and purity against a 100 bp DNA marker (Real Biotech Corporation, Taiwan). Reactions were purified and sequenced by Macrogen (Seoul, South Korea), using ABI PRISM3700 automated sequencing instrumentation (Applied Biosystems, MA, USA). New alleles and sequence types (STs) were submitted to the *Burkholderia pseudomallei* MLST database curator.

### Whole genome sequencing

*Burkholderia pseudomallei* DNA libraries were prepared for multiplexed, paired-end sequencing on the Illumina GAIIx and Illumina HiSeq sequencing platforms (Illumina, USA). Paired-end Illumina whole genome sequence data for each isolate was aligned against both chromosomes of *B*. *pseudomallei* strain K96243 [22] using BWA-MEM v0.7.5 [23]. Duplicate regions were identified and removed based on a self-alignment of the reference genome using NUCmer v3.23 [24]. Single nucleotide polymorphisms (SNPs) were called by the UnifiedGenotyper method in GATK v2.7.5 [25, 26] on the binary alignment map (BAM) file [27]. Those SNPs below a minimum depth (10x) or a minimum allele proportion (90%) were removed from subsequent analyses. These methods are wrapped by the Northern Arizona SNP Pipeline (NASP) (http://tgennorth.github.io/NASP/) and the In Silico Genotyper pipeline [28]. Sequence data generated during this study have been submitted to the Sequence Read Archive database with accession numbers listed in Table 1.

### Phylogenetic reconstruction

Three algorithms for phylogenetic reconstruction using 49,995 orthologous SNPs were utilized and compared; neighbour joining, maximum parsimony and maximum likelihood. All trees were rooted using the near relative, *Burkholderia humptydooensis* MSMB121. Analyses were performed using MEGA 6.06 [29] with 500 bootstrap replicates for each method. For the neighbour joining phylogeny, evolutionary distances were computed using the maximum composite likelihood method [30], The model selection tool implemented in MEGA 6.0 [29] was used to determine the best fitting models based on the Bayesian information criterion score. Accordingly, the general time reversible model [31] was used to estimate substitution rates for the maximum likelihood phylogeny, while a discrete gamma distribution was used to model evolutionary variation among sites. Trees were visualized using FigTree version 1.4.2 (Institute of Evolutionary Biology, University of Edinburgh). Differences in tree topologies were calculated with Compare2Trees [32]. A linear regression was performed using TempEst, however a correlation coefficient of -0.26 revealed that a molecular clock signal is not discernible on these isolates.

## Results

**Fig 2:**
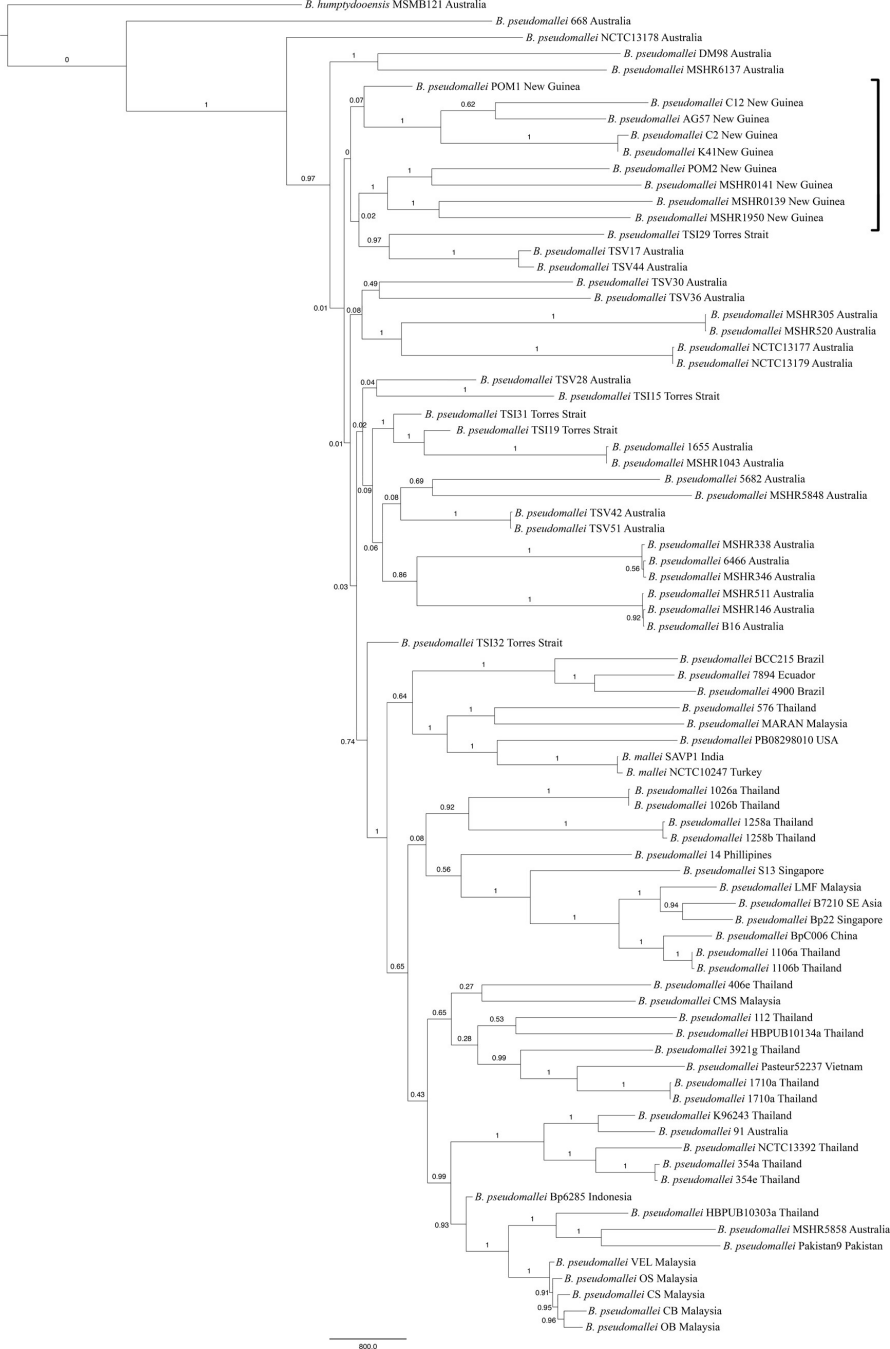
Maximum parsimony tree of *Burkholderia pseudomallei* isolates constructed using single nucleotide polymorphisms extracted from whole genome sequence data. This reconstruction demonstrates the relatedness of New Guinean and Torres Strait isolates to other *B*. *pseudomallei* isolates from around the globe. Bootstrap values (based on 500 replicates) are shown on relevant branches. New Guinean isolates are bracketed to the right of the diagram.

MLST resolved 13 sequence types across the New Guinean and Torres Strait isolates examined, with ST 267 shared by isolates C2 and K41, both from the Balimo region of New Guinea. Whole genome sequencing enabled the comparison of an effective core genome size of 1.6MB and represents high quality, non-repetitive bases that led to the discovery of 49,995 SNPs for phylogenetic inference. All three phylogenetic models (neighbour joining, maximum parsimony and maximum likelihood) produced trees that are 79.7–90.0% similar. Despite widespread topological differences, all trees agree, with strong bootstrap support (100%) deeper nodes representing the two distinct populations of *B*. *pseudomallei* that are geographically separated. Whilst isolates from Australia, New Guinea and the Torres Strait are dispersed throughout the phylograms in a paraphyletic manner, all isolates from Southeast Asia and other regions of the globe, and Australian isolates (BP91 and MSHR5858) form a single monophyletic lineage within the diverse Australian population (Fig 2). Within these major clades there are substantial homoplasies (likely a result of recombination) as indicated by low bootstrap values and a parsimony informative consistency index of 0.32 for the maximum parsimony tree.

All three models indicate that isolates from New Guinea comprise two geographically distinct clades within the Australian population with common ancestry. All isolates from Balimo (e.g., C12, AG57, C2, K41) form a single clade with excellent bootstrap support across all models. Four isolates from Port Moresby (e.g., POM2, MSHR0141, MSHR0139, MSHR1950) form the second New Guinea clade, that was supported by 100% of bootstrap iterations across all methods. The fifth Port Moresby isolate (POM1) is sister to the Balimo clade in the MP tree; however, bootstrap support for this position is poor. In contrast, the neighbour joining and maximum likelihood models suggest that this isolate is the sole representative of yet another New Guinean lineage. All trees indicate that the New Guinean clades stem from basal positions in the phylogeny; however, their exact positions differ based on phylogenetic methodology.

Likewise, the five genomes from the Torres Strait are also placed throughout the more basal parts of the tree and are not closely related to any of the other strains or in well-supported, geographic-specific clades. The exception to this is an isolate from Boigu Island (TSI15) which falls into a clade with isolates from Balimo with 100% bootstrap support in the neighbour joining and maximum likelihood trees. The maximum parsimony tree (Fig 2) shows an alternate placement with an isolate from Townsville, but with very low statistical support (4%). Again, the exact phylogenetic position and topology of many of these deep clades differ between trees and are generally not supported by high bootstrap values. Whilst bootstrap support is 100% for the major clades of Australasia and Asia, topological uncertainty within the Australasian clade is characterized by a wide range of bootstrap support for many intermediate groupings. This correlates with the findings of Pearson et al. (2009) who found that the basal topology of the *B*. *pseudomallei* tree is marked with uncertainty, probably due to rapid radiation events and ancient lateral gene transfer.

## Discussion

Prior studies [7, 10, 11] have used whole genome sequencing to confirm the presence of two distinct populations of *B*. *pseudomallei* broadly corresponding to mainland Australia and Southeast Asia, with the Australian population identified as the most ancestral population in all cases. The scarcity of genotypic or phylogenetic overlap from one population (Australasia) to the other (Southeast Asia) is a testament to the strength of ancient oceanic biogeographical barriers in limiting *B*. *pseudomallei* dissemination as previously hypothesised [7, 18].

We therefore hypothesized that pelagic regions separating other islands would similarly serve as more recent dispersal barriers for *B*. *pseudomallei* populations in the Torres Strait and New Guinea. The genetic diversity of these isolates as determined using MLST [18] and multiple monophyletic clades (from WGS analyses) from New Guinea and Torres Strait Islands suggest that multiple independent dispersal events would be needed to explain the current distribution. Rather, this distribution is most parsimoniously explained as remnants of an ancient Sahul population. Although the ancient route of *B*. *pseudomallei* dissemination across Australasia remains incompletely understood, our findings suggest that these populations may have evolved in allopatry when rising sea levels at the end of the Last Glacial Maximum approximately 19,000 years before present [14] isolated them from mainland Australia. Divergence estimates may bolster such hypothesis, however attempts to date using a linear regression model is not likely to yield informative data. This hypothesis does not exclude the possibility that limited dispersal events across pelagic boundaries also occurred, although there is currently insufficient data to support this argument.

Whilst some deep evolutionary nodes in the *B*. *pseudomallei* phylogeny are well supported among trees, others are particularly difficult to resolve, probably due to both rapid radiations as well as lateral gene transfer events. The limited population structuring among *B*. *pseudomallei* isolates on a local scale in New Guinea is likely a result of high levels of genetic admixture [7, 8, 33], but not necessarily widespread dispersal. For example, a population gradient, coupled with infrequent dispersal may also produce the population genetic structure seen in this species. Barriers to dispersal are not limited to pelagic boundaries and may also exist, albeit to a lesser extent, across the terrestrial environment. For example, a clonal *B*. *pseudomallei* population thought to have been introduced circa 1966 into southwestern Australia has persisted without additional introductions [34, 35]. Other terrestrially linked populations may have remained in isolation for much longer periods. Abnormally high rainfall in central Australia, a region previously not considered endemic for melioidosis, led to the discovery of novel and diverse lineages of *B*. *pseudomallei* not seen elsewhere in Australia or globally [36]. Even within the highly endemic region of northern Australia, McRobb et al. (2014) demonstrated the existence of geographic structuring between isolates from the Northern Territory and Queensland. Recent analysis of the distribution of STs in the Northern Territory of Australia has also supported limited dispersal of the large number of STs identified to date in that large region, with *B*. *pseudomallei* spread generally limited to a maximum distance of 45 km [37], although there is one notable exception [38]. In New Guinea, isolates from Balimo and Port Moresby fall into separate monophyletic clades, suggesting that like these other examples, dispersal and subsequent ecological establishment may be uncommon. The frequency of successful introduction events leading to ecological establishment is likely to be variable and highly dependent on geographic boundaries, modes and frequency of dispersal, proximity to endemic regions, anthropogenic factors and ecological conditions.

Human-mediated dispersal has played a critical role in the distribution of many bacterial pathogens [39–42] and continues to do so [43, 44]. There is ample evidence of anthropogenic introductions of *B*. *pseudomallei* into non-endemic regions of the world [10, 34, 45–47]. It is therefore likely that due to globalisation, human activities are now more important in influencing the population genetics of this species [48]. Human mobility and environmental impact in New Guinea is limited in comparison to other regions, and is reflected in a high diversity of culture, language and human genetics [49]. Given that the only modern modes of human transport between Balimo and Port Moresby are by air or sea, the dissemination of *B*. *pseudomallei* isolates between the regions by human activities is particularly limited. It is therefore likely that ancient *B*. *pseudomallei* populations have persisted in New Guinea relatively free of the influence of recent human trade and cultural practices. The observation that isolates from New Guinea form distinct regional clades, whereas those from mainland Australia and Southeast Asia do not conform to such constraints [2, 5], indicates that anthropogenic influences or other mechanisms facilitating dispersal of the organism across a terrestrial environment occur more frequently in other parts of Australia and Asia. Such observation may be linked to the stark contrast between nomadic lifestyles of the traditional mainland Australian people verses the relatively sedentary lifestyles of the diverse human populations in New Guinea.

The limited diversity of *B*. *pseudomallei* from the island of New Guinea in comparison to that of other regions raises questions as to role of recent anthropogenic influences on the distribution and establishment of new *B*. *pseudomallei* populations in this region, and by extension the globe. Furthermore, the observations of this study are congruent with contemporary hypotheses that longer-term separation of *B*. *pseudomallei* populations each side of the Wallace line are responsible for divergent evolution of Southeast Asian and Australasian populations of *B*. *pseudomallei* in a manner congruent with that of macro flora and fauna in the region. Robust theories of microbial biogeography therefore, must consider not only traditional biogeographical factors on geological time-scales, but account for the recent and ongoing impact of globalisation and human activity.
